# Pelvic lymphadenectomy: Step-by-step surgical education video

**DOI:** 10.4274/jtgga.galenos.2019.2018.0167

**Published:** 2020-03-06

**Authors:** İlker Selçuk, Bora Uzuner, Erengül Boduç, Yakup Baykuş, Bertan Akar, Tayfun Güngör

**Affiliations:** 1 Clinic of Gynecologic Oncology, University of Health Sciences Turkey, Ankara Dr. Zekai Tahir Burak Woman’s Health Training and Research Hospital, Ankara, Turkey; 2 Department of Anatomy, Kafkas University Faculty of Medicine, Kars, Turkey; 3 Department of Obstetrics and Gynecology, Kafkas University Faculty of Medicine, Kars, Turkey; 4 Clinic of Obstetrics and Gynecology, İstinye University, WM Medical Park Hospital, Kocaeli, Turkey

**Keywords:** Lymph node, anatomy, gynecologic oncology, lymphadenectomy, surgery

## Abstract

Pelvic lymph node dissection is one of the leading surgical procedures in gynecologic oncology practice. Learning the proper technique with anatomic landmarks will improve surgical skills and confidence. This video demonstrates a right-side systematic pelvic lymphadenectomy in a cadaveric model.

## Introduction

Pelvic lymphadenectomy is a supplementary part of staging and treatment in gynecologic oncology. Additionally, it influences the prognosis and guides the adjuvant treatment. The role of lymphadenectomy in ovarian cancer is controversial, in endometrial cancer high-risk patient groups deserve lymphadenectomy and in cervical cancer lymphadenectomy is a complementary part of the surgical treatment. Lymphadenectomy can be performed in a selective or systematic approach. This video demonstrates a right-side systematic pelvic lymphadenectomy in a cadaveric model.

### Pelvic lymphatic drainage

Drainage from the lymphatics of the perineum, lower extremities, lower abdominal wall, and pelvic viscera (except the sigmoid colon) is to the pelvic wall lymph chain. The upper paracervical (supraureteral paracervical) and lower paracervical (infraureteral paracervical) pathways are the basic routes of pelvic lymphatic drainage (1). Additionally, a lymphatic branch from the ovary runs downward from the utero-ovarian ligament and follows ovarian-uterine artery branch, consequently draining via the upper paracervical pathway (2).

The pelvic lymph nodes mainly include the external iliac, internal iliac, and obturator lymph nodes, which are below the bifurcation of the common iliac artery. The lymphatic tissues lay on the external iliac vessels anteriorly and medially, over the internal iliac vessels, at the interiliac junction, and over the obturator nerve; these lymph nodes should be removed in order to achieve a complete (systematic) pelvic lymphadenectomy (3).

The borders of the pelvic lymph nodes are the genitofemoral nerve laterally, bifurcation of the common iliac artery cranially, the deep circumflex iliac vein caudally, the obturator nerve inferiorly, and the obliterated umbilical artery medially (4).

Sacral lymph nodes are generally not encountered in the pelvic lymph node group and its dissection is not a routine part of pelvic lymphadenectomy. Sentinel lymph node mapping studies also showed that sentinel lymph nodes are rarely detected in the presacral area (5); however, if there is a bulky lymph node it should be dissected.

### Surgical technique

**Figure 1:**
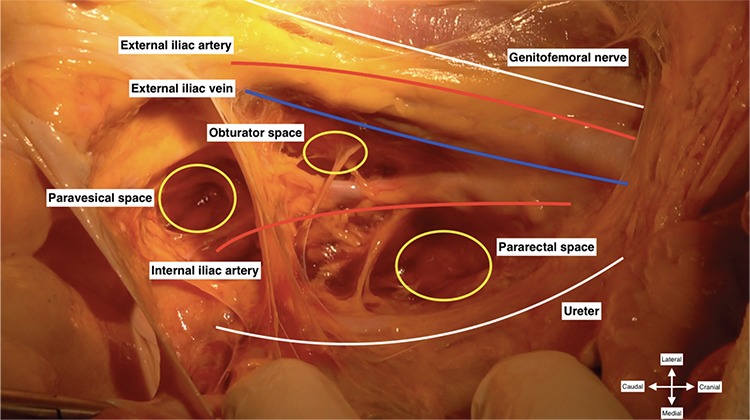
Pelvic anatomy for proper pelvic lymphadenectomy (right pelvic side wall)

In order to achieve a successful pelvic lymphadenectomy, first, a good anatomic exposure should be maintained to visualize the entire surgical field (Figure 1), secondly, lymph nodes over the external and internal iliac vessels are dissected and then the obturator lymph nodes are removed. 

After exploring the abdomino-pelvic cavity, the uterus is drawn over to the contralateral pelvic side wall, caudo-medially. The lateral parietal peritoneum is incised between the round ligament and the infundibulopelvic ligament, so the retroperitoneal space is accessed (transection of the round ligament to access the retroperitoneal area is optional). The incision is enlarged and the peritoneum is cut parallel to the infundibulopelvic ligament. The ureter is identified at the base of the posterior sheet of the broad ligament. The pararectal space is developed between the internal iliac artery (lateral) and ureter (medial). The paravesical space is developed between the bladder (medial) and pelvic side wall (lateral); the obliterated umbilical artery divides the paravesical space into two parts and the lateral part indicates the obturator fossa. The peritoneal tissue of the round ligament where it enters the inguinal canal under the inguinal ligament is pulled upward. The ureter is retracted medially, and the pelvic lymphadenectomy starts over the external iliac artery, below the bifurcation of common iliac artery.

**Figure 2:**
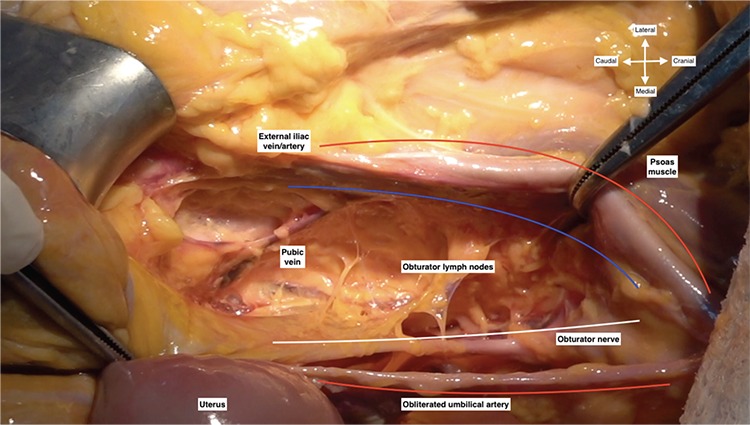
Pubic vein and obturator lymph nodes (right pelvic side wall)

The fibroadipose lymphatic tissue over the external iliac artery is gently elevated and mobilized medially, and a tiny dissection is performed to separate the lymphatic tissue from the fibrous sheath. While dissection is performed longitudinally over the external iliac artery, a cleavage is opened at the mid-level to clear the lymphatic tissue over the external iliac vein until the level of deep circumflex iliac vein, which is the caudal border. Therefore, internal iliac lymph nodes are also removed over the anterior part of internal iliac artery. Afterwards, lymph node dissection turns around the superior pubic ramus of the pubic bone, which forms a part of the obturator foramen, and the pubic vein, the connection between the external iliac and obturator vein (corona mortis), is identified. The dissection of obturator lymph nodes starts from this point after retraction of external iliac vessels laterally to the psoas muscle and maintaining a medial retraction on the paravesical space, which retracts the obliterated umbilical artery medially (Figure 2). All the lymphatic tissue over the obturator nerve lateral to the obliterated umbilical artery is stripped from the attachments and finally the lymphatic tissue is removed.

**Figure 3:**
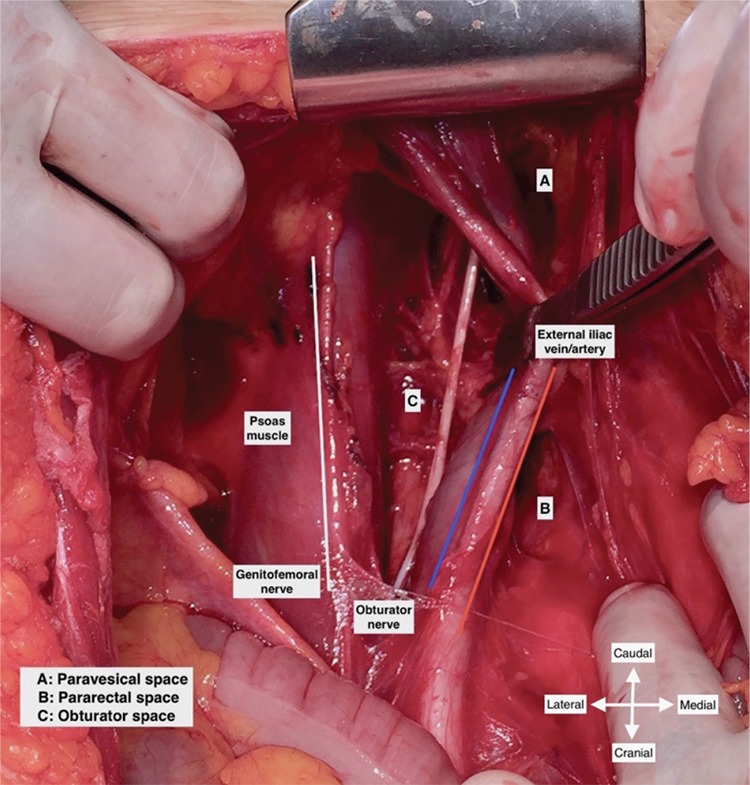
Exposure of obturator space after medial retraction of external iliac vessels (left pelvic side wall/live surgery-pelvic lymphadenectomy)

Additionally, if there is a bulky or huge lymph node over the external iliac vessels and extending to the psoas muscle, the external iliac vessels are separated from the psoas muscle by sharp and blunt dissection so the medial part of psoas muscle and obturator internus muscle can be exposed (Figure 3). This maneuver also provides a lateral access to the obturator fossa and the obturator nerve can easily be identified by applying gentle traction on the obturator lymph nodes (3,4,6).

### Probable surgical complications

- Bleeding

External iliac vessels, internal iliac vessels, obturator vessels, or pubic vein or artery (corona mortis);

- Nerve injury

Genitofemoral nerve, obturator nerve;

- Ureter injury

- Lymphorrhea

- Lymphedema

### Tips and tricks for pelvic lymphadenectomy

- Starting lymph node dissection over the anterior surface of external iliac artery is a safe method in creating the right cleavage.

- If there is a bulky lymph node at the obturator fossa and lying under the obturator nerve, care should be taken during stripping the attachments under the obturator nerve. There is an extensive venous vascular bed and collateral circulation of internal iliac vein, which makes the control of bleeding difficult (7).

- If corona mortis is formed by a pubic vein (most frequent type), the bleeding can easily be controlled (8).

- Obturator nerve injuries are rare; however, end-to-end anastomosis can be performed or nerve grafts may be applied (9).

- Ureter injuries are managed according to the region of injury; double-J-stents or end-to-end anastomosis are the options.

- The distal part of the external iliac or obturator lymph nodes over the deep circumflex iliac and pubic vein can be clipped or sutured to prevent lymphorrhea (4).

- Any self-retaining retractor may provide adequate gross exposure; however, caudomedial retraction from the paravesical space using a Deaver retractor and lateral retraction of external iliac vessels by a vessel retractor are critical points.

- Monopolar or bipolar cautery or a Metzenbaum scissor are used in dissection. Additionally, any other vessel sealing device can be used according to the preferences of the surgeon.

**Video 1.** 10.4274/jtgga.galenos.2019.2018.0167.video1
